# Melatonin is a potential drug for the prevention of bone loss during space flight

**DOI:** 10.1111/jpi.12594

**Published:** 2019-07-19

**Authors:** Mika Ikegame, Atsuhiko Hattori, Makoto J. Tabata, Kei‐ichiro Kitamura, Yoshiaki Tabuchi, Yukihiro Furusawa, Yusuke Maruyama, Tatsuki Yamamoto, Toshio Sekiguchi, Risa Matsuoka, Taizo Hanmoto, Takahiro Ikari, Masato Endo, Katsunori Omori, Masaki Nakano, Sayaka Yashima, Sadakazu Ejiri, Toshiki Taya, Hiroshi Nakashima, Nobuaki Shimizu, Masahisa Nakamura, Takashi Kondo, Kazuichi Hayakawa, Ichiro Takasaki, Atsushi Kaminishi, Ryosuke Akatsuka, Yuichi Sasayama, Takumi Nishiuchi, Masayuki Nara, Hachiro Iseki, Vishwajit S. Chowdhury, Shigehito Wada, Kenichi Ijiri, Toshio Takeuchi, Tohru Suzuki, Hironori Ando, Kouhei Matsuda, Masanori Somei, Hiroyuki Mishima, Yuko Mikuni‐Takagaki, Hisayuki Funahashi, Akihisa Takahashi, Yoshinari Watanabe, Masahiro Maeda, Hideaki Uchida, Akio Hayashi, Akira Kambegawa, Azusa Seki, Sachiko Yano, Toru Shimazu, Hiromi Suzuki, Jun Hirayama, Nobuo Suzuki

**Affiliations:** ^1^ Graduate School of Medicine, Dentistry and Pharmaceutical Sciences Okayama University Okayama Japan; ^2^ College of Liberal Arts and Sciences Tokyo Medical and Dental University Ichikawa Japan; ^3^ Graduate School of Tokyo Medical and Dental University Bunkyo‐ku Japan; ^4^ Faculty of Health Sciences, Institute of Medical, Pharmaceutical and Health Sciences Kanazawa University Kodatsuno Japan; ^5^ Life Science Research Center University of Toyama Toyama Japan; ^6^ Department of Liberal Arts and Sciences Toyama Prefectural University Toyama Japan; ^7^ Division of Marine Environmental Studies, Noto Marine Laboratory, Institute of Nature and Environmental Technology Kanazawa University Noto‐cho Japan; ^8^ Department of Marine Biosciences Tokyo University of Marine Science and Technology Minato‐ku Japan; ^9^ Faculty of Economics Asia University Musashino Japan; ^10^ Division of Oral Structure, Function and Development Asahi University School of Dentistry Mizuho Japan; ^11^ Agilent Technologies Japan Ltd. Hachioji Japan; ^12^ Faculty of Education and Integrated Arts and Sciences Waseda University Shinjuku‐ku Japan; ^13^ Graduate School of Medicine and Pharmaceutical Sciences University of Toyama Toyama Japan; ^14^ Low Level Radioactivity Laboratory, Institute of Nature and Environmental Technology Kanazawa University Nomi Japan; ^15^ Graduate School of Science and Engineering University of Toyama Toyama Japan; ^16^ Institute for Gene Research, Advanced Science Research Center Kanazawa University Kanazawa Japan; ^17^ Faculty of Arts and Science Kyushu University Fukuoka Japan; ^18^ Faculty of Medicine University of Toyama Toyama Japan; ^19^ Radioisotope Center University of Tokyo Bunkyo‐ku Japan; ^20^ Graduate School of Agricultural Science Tohoku University Sendai Japan; ^21^ Marine Biological Station, Sado Center for Ecological Sustainability Niigata University Sado Japan; ^22^ Laboratory of Regulatory Biology, Graduate School of Science and Engineering University of Toyama Toyama Japan; ^23^ Department of Dental Engineering Tsurumi University School of Dental Medicine Yokohama Japan; ^24^ Kanagawa Dental University Graduate School of Dentistry Yokosuka Japan; ^25^ Department of Physical Therapy, Faculty of Makuhari Human Care Tohto University Mihama‐ku Japan; ^26^ Gunma University Heavy Ion Medical Center Maebashi Japan; ^27^ Organization of Frontier Science and Innovation Kanazawa University Kanazawa Japan; ^28^ Molecular Devices Japan Ltd. Chuo‐ku Japan; ^29^ Kambegawa Laboratory Komae Japan; ^30^ HAMRI Co. Ltd. Koga Japan; ^31^ Japan Aerospace Exploration Agency Tsukuba Japan; ^32^ Japan Space Forum Chiyoda‐ku Japan; ^33^ Department of Clinical Engineering, Faculty of Health Sciences Komatsu University Komatsu Japan

**Keywords:** calcitonin, fish scales, microgravity, osteoblasts, osteoclasts, RANKL

## Abstract

Astronauts experience osteoporosis‐like loss of bone mass because of microgravity conditions during space flight. To prevent bone loss, they need a riskless and antiresorptive drug. Melatonin is reported to suppress osteoclast function. However, no studies have examined the effects of melatonin on bone metabolism under microgravity conditions. We used goldfish scales as a bone model of coexisting osteoclasts and osteoblasts and demonstrated that mRNA expression level of *acetylserotonin O‐methyltransferase*, an enzyme essential for melatonin synthesis, decreased significantly under microgravity. During space flight, microgravity stimulated osteoclastic activity and significantly increased gene expression for osteoclast differentiation and activation. Melatonin treatment significantly stimulated *Calcitonin* (an osteoclast‐inhibiting hormone) mRNA expression and decreased the mRNA expression of *receptor activator of nuclear factor κB ligand* (a promoter of osteoclastogenesis), which coincided with suppressed gene expression levels for osteoclast functions. This is the first study to report the inhibitory effect of melatonin on osteoclastic activation by microgravity. We also observed a novel action pathway of melatonin on osteoclasts via an increase in CALCITONIN secretion. Melatonin could be the source of a potential novel drug to prevent bone loss during space flight.

## 1 INTRODUCTION

Exposure to microgravity during space flight leads to rapid bone loss in humans and animals. The rapid and vigorous bone loss is one of the key challenges that remain to be solved to enable humans to pursue healthy lives in outer space. Bone mass often reflects a balance between bone formation by osteoblasts and bone resorption by osteoclasts. An increasing number of studies have demonstrated the effects of microgravity on bone metabolism and osteoblast activity, and increased bone resorption under microgravity has been widely reported.^1^, ^2^, ^3^, ^4^, ^5^ However, the cellular and molecular mechanisms underlying the phenomena remain unclear. Therefore, further investigations on the mechanisms that could facilitate the discovery of medical drug that effectively addresses bone loss in space are required.

In bone tissue, differentiation and activation of osteoclasts are generally influenced by interactions with osteoblastic lineage cells.^6^, ^7^ Osteoclasts express the receptor activator for nuclear factor *κ*B (RANK), which is a receptor for the RANK ligand (RANKL). RANK ligand is expressed in some stromal cells such as osteoblasts and bone marrow cells, and is required for osteoclastogenesis and osteoclast activation.^6^, ^7^ RANKL signaling is inhibited by osteoprotegerin (OPG), which is a decoy receptor produced by stromal cells, and the ratio of RANKL:OPG expression is critical for osteoclastogenesis.^6^ Therefore, interactions between osteoclasts and stromal cells are essential for evaluating the mechanisms underlying rapid bone loss during space flight. Although coculture experiments of isolated osteoblasts and osteoclasts are feasible, they do not accurately reflect natural cellular microenvironments. Therefore, the lack of appropriate experimental models for interactions between osteoclasts and stromal cells implies that no published data currently demonstrate the precise mechanisms underlying bone loss during space flight.

Teleost scales are calcified tissues that exhibit multiple similarities with mammalian membrane bone tissues.^8^, ^9^ Morphological features of goldfish scales are illustrated in Figure S1. Fish scales can regenerate following scale removal. The regenerating scales contain multinucleated osteoclasts with actin rings, well‐developed ruffled borders, and clear zones. Active cuboidal osteoblasts are observed at the periphery and on the dermis side of the scale. They represent a highly developed rough endoplasmic reticulum and a Golgi apparatus. In addition, biological responses to mechanical stimuli are maintained in regenerating scales after storage at 4°C for 1 week.^10^ Therefore, regenerating scales are the most suitable materials for a space experiment examining interactions between osteoblasts and osteoclasts.

Antiresorptive drugs, such as bisphosphonates, are expected to prevent bone loss during space flight.^11^ However, some antiresorptive drugs cause unfavorable side effects in some cases such as osteonecrosis of the jaw.^12^ Therefore, riskless drugs are required to prevent bone loss during space flight. Melatonin is the principal secretory product of the pineal gland and is synthesized almost exclusively in the dark. Therefore, its blood levels exhibit a discernible daily rhythm.^13^ In 2002, we reported, for the first time, that melatonin suppresses osteoclast activity using a goldfish scale culture system.^14^ The report predicted the potential application of melatonin as a therapeutic drug for bone loss. Furthermore, following surgical ablation of the pineal gland, spinal malformations have been observed in Atlantic salmon, chicks, and rats.^9^ In humans, bone mineral density in the femoral neck of postmenopausal osteopenic women increased in response to melatonin in a dose‐dependent manner when compared with a placebo.^15^ In addition, in melatonin‐treated perimenopausal women, the ratio of bone resorption marker:formation marker exhibited a decreasing trend.^16^ The results indicate that treatment with melatonin is a superior method for suppressing osteoclast activity under microgravity conditions, in addition to on the ground. It was recently reported that melatonin is produced in the brain, retina, intestine, ovary, skin, and crystalline lens, which implies that melatonin could have widespread physiological functions in numerous tissues.^17^

Investigating whether melatonin increases an osteoclast‐inhibiting hormone, CALCITONIN, in goldfish scales and whether the increased CALCITONIN regulates osteoclast function via paracrine system could offer insights on the role of melatonin. The present study performed space experiments to analyze bone metabolism in goldfish scale during space flight to determine the melatonin‐dependent regulatory mechanisms of osteoclasts.

## 2 METHODS

### 2.1 Goldfish

Goldfish (*Carassius auratus*) specimens were purchased from Higashikawa Fish Farm (Yamatokoriyama, Japan) and were artificially fertilized at Tokyo University of Marine Science and Technology. Experiments with growing fish (10‐12 cm) were performed according to the recommendations in the ethical guidelines of Kanazawa University and Tokyo Medical and Dental University.

### 2.2 Comparison of melatonin production between ontogenic and regenerating goldfish scales

Regenerating scales on day 14 were obtained from goldfish.^18^, ^19^ The samples from the ontogenic and the regenerating scales were subjected to liquid chromatography coupled with tandem mass spectrometry (LC‐MS/MS) as illustrated in Figure S2.

### 2.3 Space flight experiment

The regenerating scales were packed into culture chambers (60 scales in each chamber) and 96‐well plates (1 scale in each well) with and without melatonin (1 μM; Sigma‐Aldrich Co. LLC) as illustrated in Figure S3 and flown on the space shuttle Atlantis flight STS‐132 (NASA, USA) as illustrated in Figure S4. After arrival at Japan's space laboratory (KIBO) at the International Space Station (ISS), one 96‐well plate was frozen at −95°C as a launch control for enzyme activity analysis. Before incubation, the other 96‐well plates and the culture chambers were kept at 4°C and then incubated for 86 hours at 22°C in in‐flight microgravity (F‐μg) or in‐flight artificial 1 gravity (F‐1g) at the Cell Biology Experiment Facility^20^ (Videos S1 and S2).

Subsequently, the scales in 96‐well plates were frozen at −95°C for tartrate‐resistant acid phosphatase (TRAP) activity analysis.^18^ The culture medium was replaced with RNAlater (Sigma‐Aldrich) for gene expression analysis or with 4% paraformaldehyde (PFA) phosphate buffer for morphological analysis (Videos S1 and S2). Specimens were stored at −95°C for gene expression analysis or at 4°C for morphological analysis until the return of STS‐132 to the Kennedy Space Center in Florida.

Some scales were incubated with melatonin (1 μM) in F‐μg conditions and processed for gene expression analysis.

### 2.4 Histological analyses

The scales fixed with 4% PFA were incubated in 0.1% Triton X‐100 for 10 minutes. They were then used for actin ring formation and TRAP activity analyses^21^ (Figure S1).

For the immunohistochemical analysis, the fixed scales were used as whole‐mount samples or cryosections. They were incubated with a blocking solution and then with primary antibodies: anti‐acetylserotonin O‐methyltransferase (ASMT) (ab180511; Abcam; ×100), anti‐CALCITONIN (×50 000),^22^ or anti‐melatonin receptor (anti‐Mel‐R) (MTNR1B/MT2 antibody; LS‐A934; LifeSpan BioSciences; ×1000). For the secondary antibody, Alexa Fluor® 488‐labeled anti‐rabbit IgG (A11034, Molecular Probes; ×1000) was used. For the negative controls, immunostaining was also performed using anti‐CALCITONIN or anti‐Mel‐R that had been pre‐absorbed with 5 μg/mL of salmon CALCITONIN or 2 μg/mL of peptide for Mel‐R (LS‐E29610; LifeSpan BioSciences), respectively.

For in situ hybridization, digoxigenin (DIG)‐labeled sense and antisense single‐stranded RNA probes for goldfish *Asmt* were prepared using a cDNA fragment containing 324‐1244 bases in GU205783. The signals of specific transcripts were detected using a kit (Boehringer Mannheim). The sections were counterstained with methyl green.

### 2.5 Histomorphometry

Regenerating scales had enlarged foci with complex mesh‐like networks of grooves (Figure S1B). In the histomorphometric analysis, the areas observed were restricted to regions with enlarged foci that were not covered with epithelia. For the space flight experiment, six (for F‐μg) or eight (for F‐1g) scales were randomly selected and histomorphometric analysis was performed on six 0.31 mm^2^ observation areas. Mean measurements from the six areas were regarded as representative values for each scale.

The numbers of the osteoclasts per mm^2^ were recorded, and the mean number of nuclei per multinucleated osteoclast and the percentage of osteoclasts with actin rings were calculated. The width of the grooves at the middle of each groove, actin ring size, and the percentage of groove lengths covered with actin rings were measured using NIH Image J (https://imagej.nih.gov/ij/).

### 2.6 Quantitative real‐time PCR

Total RNA isolation and cDNA synthesis were performed using a kit (Qiagen GmbH).^23^ Primers used for quantitative real‐time PCR (qPCR) are listed in Table S1. A TaqMan probe was used for *Calcitonin* (Figure S5). *Elongation factor 1 alpha* (*Ef1α*) was used for the standardization procedure.^24^

### 2.7 Reanalysis of data in an open public repository

To compare *arylalkylamine N‐acetyltransferase* (*Aanat*) and *acetylserotonin O‐methyltransferase* (*Asmt*) expression levels between space flight and ground control conditions in medaka brain samples, raw fastq files of RNA‐sequencing (RNA‐seq) data were downloaded from the DNA Data Bank of Japan database (accession no. DRA003542).^25^ De novo assembly using Trinity, mapping using Bowtie2, annotation using BLASTN, and normalization using the fragments per kilobase of exon per million mapped reads method were performed. Among the *Aanat* and *Asmt* candidates, the longest transcripts that exhibited the highest homologies (*Aanat*: 4506 bp/85.25%; *Asmt*: 1333 bp/99.86%) were predicted to be the promising transcripts in this study.

### 2.8 Effects of melatonin on CALCITONIN production in cultured regenerating goldfish scales and rat calvariae on the ground

The regenerating scales were incubated in a culture medium containing 10 nM to 1 μM of melatonin for 1, 2, and 4 days. Some scales were incubated with Mel‐R antagonist luzindole (10 μM; Tokyo Chemical Industry, Co., Ltd.), and melatonin (1 μM) for 4 days. In addition, the CALCITONIN concentrations in the media were measured using ELISA for salmon CALCITONIN antibody^22^ and for rat CALCITONIN antibody (Peninsula Laboratories International Inc).

To examine the effects of melatonin in mammalian bones, 10 calvariae excised from 2‐day‐old Wistar rats were cut in half, and two of the calvaria halves were incubated in a well with BGJb medium (Gibco BRL, Life Technologies, Inc; 10% FCS, antibiotics) containing melatonin (1 µM) or vehicle at 37°C for 1 day. The CALCITONIN concentrations in the culture media (n = 5 for each group) were measured as described above. Experiments with rats were performed according to the recommendations in the ethical guidelines of Okayama University.

### 2.9 Statistical analyses

Student's *t* test or paired *t* test was performed to analyze significant differences between the two groups. In addition, one‐way repeated‐measures analysis of variance (ANOVA) or one‐way ANOVA followed by Tukey's post hoc test was used to examine significant differences in the values among three or four groups.

## 3 RESULTS

### 3.1 Comparison of melatonin levels between ontogenic and regenerating scales

**Figure 1:**
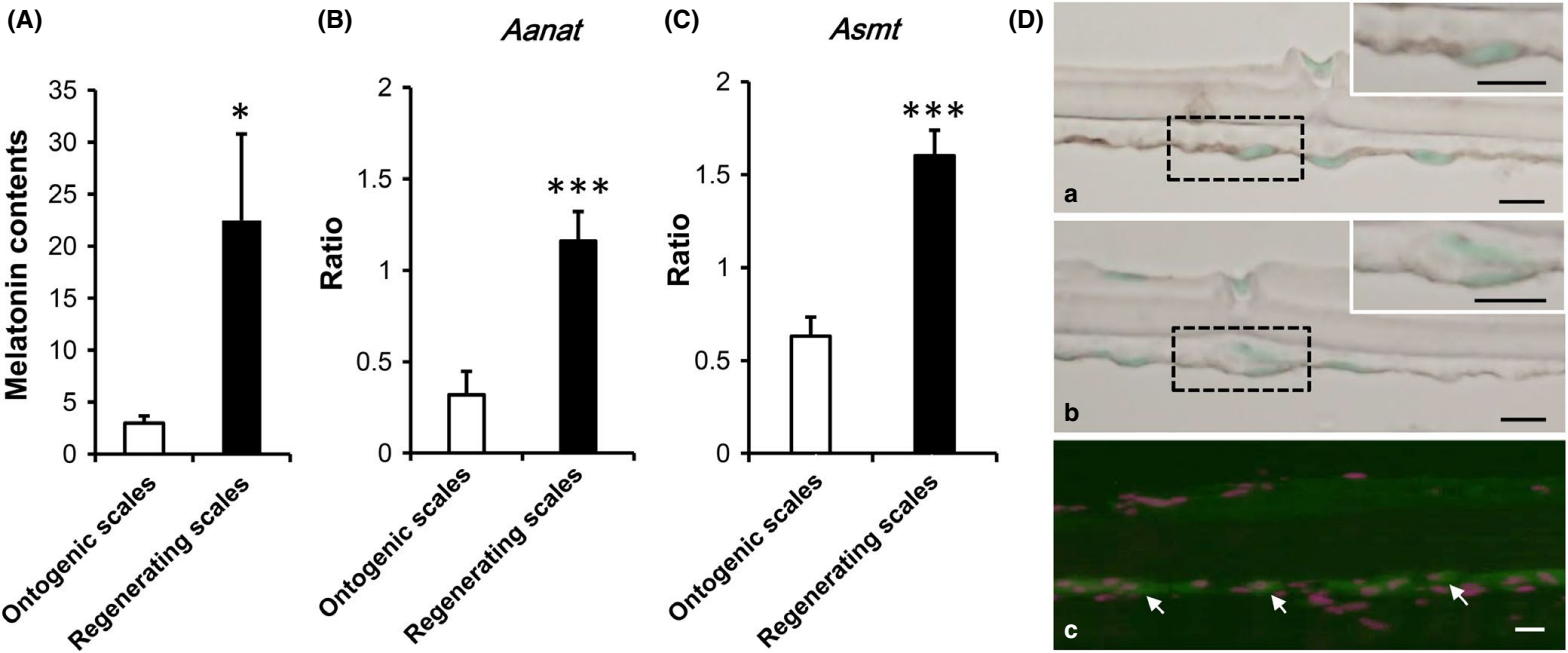
Melatonin production in ontogenic and regenerating scales of goldfish. A, The melatonin contents (pg/scale weight [g]) of ontogenic and regenerating scales were analyzed using LC‐MS/MS (each n = 12). B, C, Expression levels of *arylalkylamine N‐acetyltransferase* (*Aanat*) (B) and *acetylserotonin O‐methyltransferase* (*Asmt*) (C) examined using real‐time PCR (each n = 6). All targets were standardized to *elongation factor 1 alpha* (*Ef 1α*) expression levels and presented as relative ratios. D, *Asmt* mRNA expression in the regenerating scales was detected by in situ hybridization (a) and ASMT protein by immunohistochemistry (c) on cryosections. a: *Asmt* antisense probe, b: *Asmt* sense probe. The parts surrounded by the dotted lines were magnified and shown in the upper right panels. The signals are dark brown. c: anti‐ASMT antibody. Arrows indicate ASMT‐expressing cells (green) on the fibrillary plate. The nucleus was stained with DAPI (4', 6‐diamidino‐2‐phenylindole) (magenta). The images are representative data obtained from repeated experiments using five or more scales from different goldfish. All data are mean ± SEM; *P < .05, ***P < .001; scale bars, 10 μm

The extracts from both ontogenic and regenerating scales were subjected to LC‐MS/MS (Figure S2). The melatonin contents in the regenerating scales were significantly higher than those in the ontogenic scales (Figure 1A). Both the *Aanat* and *Asmt* mRNA levels in the regenerating scales were also significantly higher than those in the ontogenic scales (Figure 1B,C). *Asmt* mRNA (Figure 1Da,Db) and the protein (Figure 1Dc) were also detected in osteoblasts on the fibrillary plate.

### 3.2 Comparison of melatonin‐related enzyme expression in regenerating scales cultured on the ground with those cultured in space

**Figure 2:**
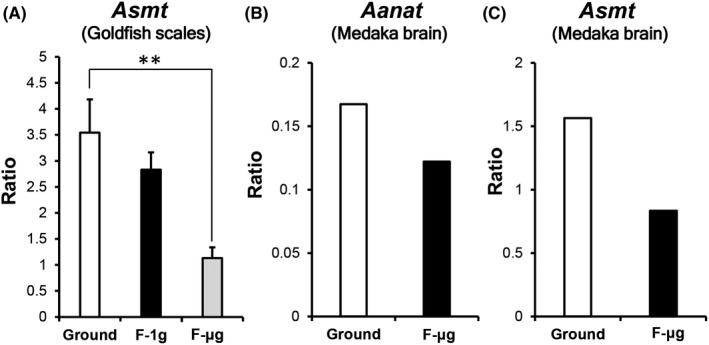
Comparison of melatonin‐synthesizing enzymes' expression levels in regenerating goldfish scales cultured on the ground against those cultured in outer space. A, *Asmt* mRNA expression levels in the regenerating scales during space flight in artificial 1 gravity (F‐1g) or microgravity (F‐µg), and on the ground analyzed using quantitative real‐time PCR. All targets were standardized to *Ef1α* expression levels and presented as relative ratios (each n = 4). B, C, *Aanat* (B) and *Asmt* (C) mRNA levels in the brains of medaka kept on the ground or in microgravity conditions during space flight. Data from the DNA Data Bank of Japan database were used.^25^ Data (A) are mean ± SEM; **P < .01

The *Asmt* mRNA expression levels in F‐μg scales decreased significantly when compared with those on the ground (Figure 2A). The *Aanat* expression levels, which were measured in goldfish scales on the ground (Figure 1B), were not detected in both F‐μg and F‐1g scales in the present stage. We aligned the raw sequence data with the reference sequences in medaka brain^25^ based on de novo assembly to quantify medaka *Aanat* and *Asmt* expression levels and found that they decreased during space flight (Figure 2B,C).

### 3.3 Osteoclast multinucleation and resorption activity in regenerating scales during space flight

**Figure 3:**
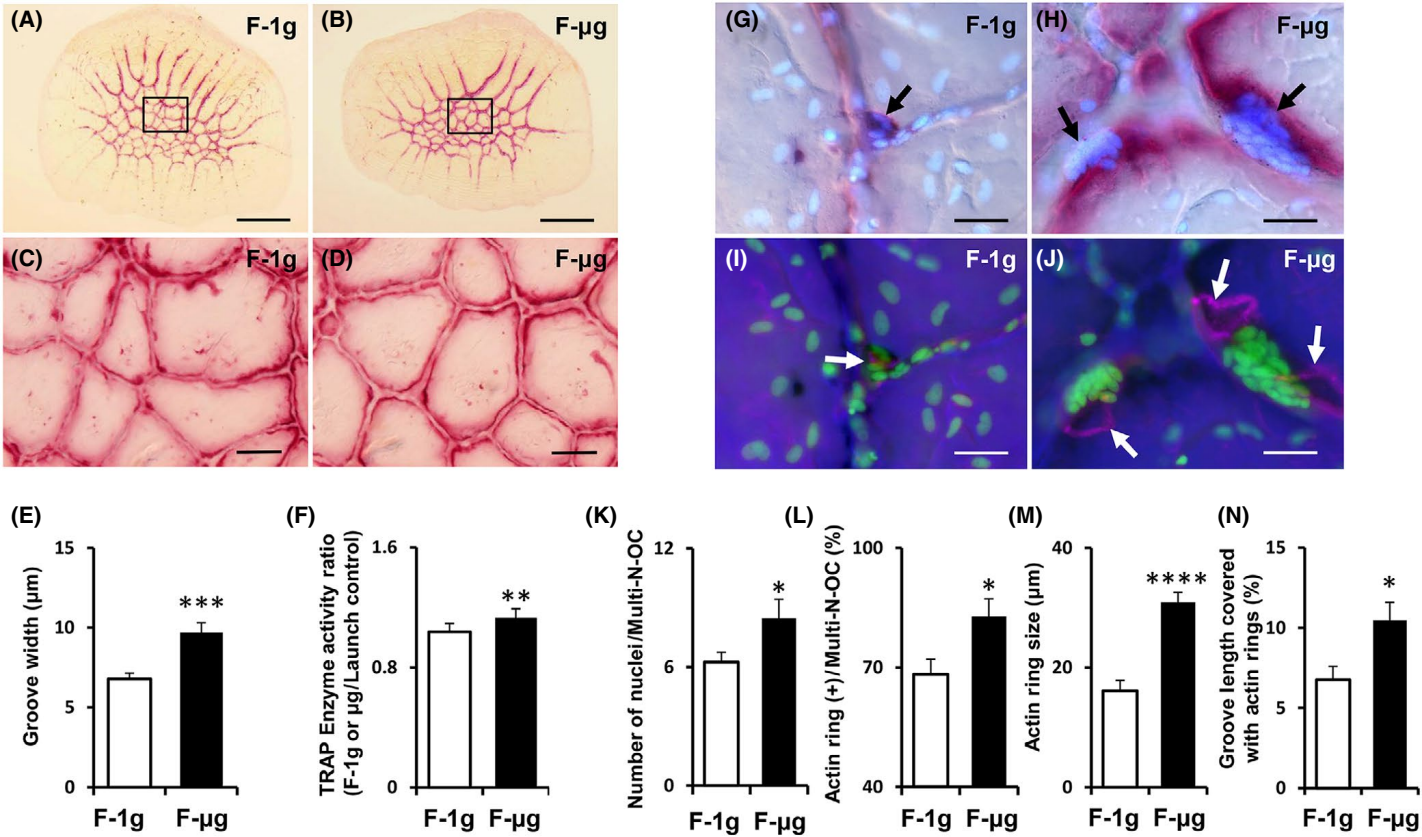
Morphological characteristics of regenerating goldfish scales during space flight. A‐D, Light microscopy images of regenerating scales stained for TRAP activity cultured during space flight in artificial 1 gravity (F‐1g) or microgravity (F‐μg). Low‐magnification observations of whole scales (A, B); higher magnifications of the square areas in A and B, respectively (C, D). TRAP staining (red) was concentrated along the edges of the grooves. E, Impact of microgravity on the groove width. F, The TRAP activity ratio between the launch control and F‐1g or F‐μg scales. G‐J, Light and fluorescence microscopy images of osteoclasts (G and I, F‐1g; H and J, F‐μg). G, H, TRAP staining (red) superposed on DAPI staining (blue) of the nuclei. The multinucleated osteoclasts (black arrows) exhibited high TRAP activity. I, J, F‐actin staining (magenta) superposed on DAPI staining (green) of the nuclei. The actin rings (white arrows) of multinucleated osteoclasts were located along the grooves. K, The average number of nuclei per multinucleated osteoclast. L, The percentage of multinucleated osteoclasts with actin rings. M, The average actin ring size measured as the diameter of the actin ring along the groove. N, The percentage of the groove length covered by actin rings. Precise measurement methods were described in the “Methods for histomorphometry” of Supplemental information (last page). All data are mean ± SEM; n = 8 for F‐1g and n = 6 for F‐μg; *P < .05, **P < .01, ***P < .001, ****P < .0001; scale bars, 1 mm in A and B, 100 μm in C and D, 20 μm in G to J

TRAP activity was mainly observed along the edges of grooves in the scales subjected to F‐μg and F‐1g (Figure 3A,B). The groove widths were significantly greater in F‐μg scales than in F‐1g scales (Figure 3C‐E). Furthermore, TRAP activity significantly increased in F‐μg scales when compared with activity in F‐1g scales (Figure 3F).

The sizes of osteoclasts seemed larger, and the number of nuclei in multinucleated osteoclasts was significantly greater in F‐μg scales than in F‐1g scales (Figure 3G,H,K). However, the total nuclear number of mono‐ and multinucleated osteoclasts exhibited no significant difference between the F‐μg and F‐1g groups (Figure S6).

The percentage of actin ring‐positive multinucleated osteoclasts and the actin ring size were significantly greater in the F‐μg scales (Figure 3I,J,L,M) than in the F‐1g scales. In addition, since actin rings were mostly observed along scale grooves, the percentage of lengths of grooves associated with the actin rings was higher in F‐μg scales than in the F‐1g scales. (Figure 3N).

### 3.4 Effects of microgravity and melatonin on bone‐related genes' expression in regenerating scales

**Figure 4:**
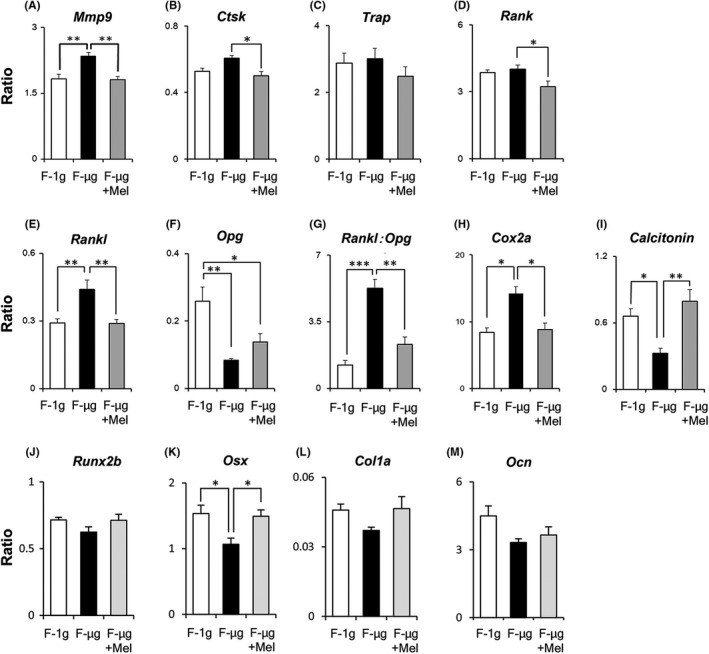
The effect of melatonin on mRNA expression of osteoclast and osteoblast‐related factors in regenerating goldfish scales during space flight. A‐I, mRNA expression levels of osteoclast‐related factors: *matrix metalloproteinase‐9* (*Mmp‐9*) (A), *cathepsin K* (*Ctsk*) (B), *tartrate‐resistant acid phosphatase* (*Trap*) (C), *receptor activator of nuclear factor κβ* (*Rank*) (D), *receptor activator of nuclear factor κβ ligand* (*Rankl*) (E), *osteoprotegerin* (*Opg*) (F), ratios of *Rankl*:*Opg* mRNA expression (G), *cyclooxygenase‐2a* (*Cox2a*) (H), and *Calcitonin* (I). J‐M, mRNA expression levels of osteoblast‐related factors: *runt‐related transcription factor 2b* (*Runx2b*) (J), *osterix* (*Osx*) (K), *type I collagen* *1a* (*Col1a*) (L), and *osteocalcin* (*Ocn*) (M). F‐1g, flight 1g; F‐μg, flight microgravity; F‐μg + Mel, F‐μg treated with melatonin (1 μM). All targets were standardized to *Ef1α* expression levels and presented as relative ratios. All data are mean ± SEM (n = 4); *P < .05, **P < .01, ***P < .001

Among the factors stimulating matrix resorption, expression level of *matrix metalloproteinase‐9* (*Mmp‐9*) significantly increased in F‐μg condition (Figure 4A), while the levels of *cathepsin K* (*Ctsk*; Figure 4B) and *Trap* (Figure 4C) exhibited an upward trend in F‐μg condition, compared with those in F‐1g condition. Microgravity significantly stimulated the expression of *Rankl* (Figure 4E) but significantly suppressed the expression of *Opg* (Figure 4F), which led to a remarkable increase in the ratio of *Rankl*:*Opg* (Figure 4G). In addition, the expression of *cyclooxygenase‐2a* (*Cox2a*) significantly increased under F‐μg condition (Figure 4H). In contrast, the expression of *Calcitonin*, whose translated product has inhibitory effects on osteoclast activity,^26^, ^27^ reduced significantly in F‐μg condition compared with that in F‐1g condition (Figure 4I).

Notably, melatonin treatment changed the expression levels of the genes analyzed to levels comparable with those in F‐1g. Melatonin significantly suppressed the increase in the expression of genes for the osteoclastogenic factors in F‐μg (Figure 4A,B,E,G,H). Conversely, it increased the expression levels of osteoclast inhibitory factors: *Opg* and *Calcitonin* (Figure 4F,I). In addition, melatonin significantly reduced the expression level of the osteoclast marker *Rank* (Figure 4D).

The expression levels of osteoblastic factors—*runt‐related transcription factor 2b* (*Runx2b*), *osterix* (*Osx*), *type 1 collagen 1a* (*Col1a*), and *osteocalcin* (*Ocn*)—were lower in F‐μg scales than in F‐1g scales (Figure 4J‐M). In the case of *Osx*, a significant difference was observed between F‐μg and F‐1g scales (Figure 4K). Melatonin treatment maintained the expression levels of *Runx2b*, *Osx*, and *Col1a* at F‐1g levels (Figure 4J‐L).

### 3.5 Effects of melatonin on CALCITONIN production in cultured regenerating scales and rat calvariae on the ground

**Figure 5:**
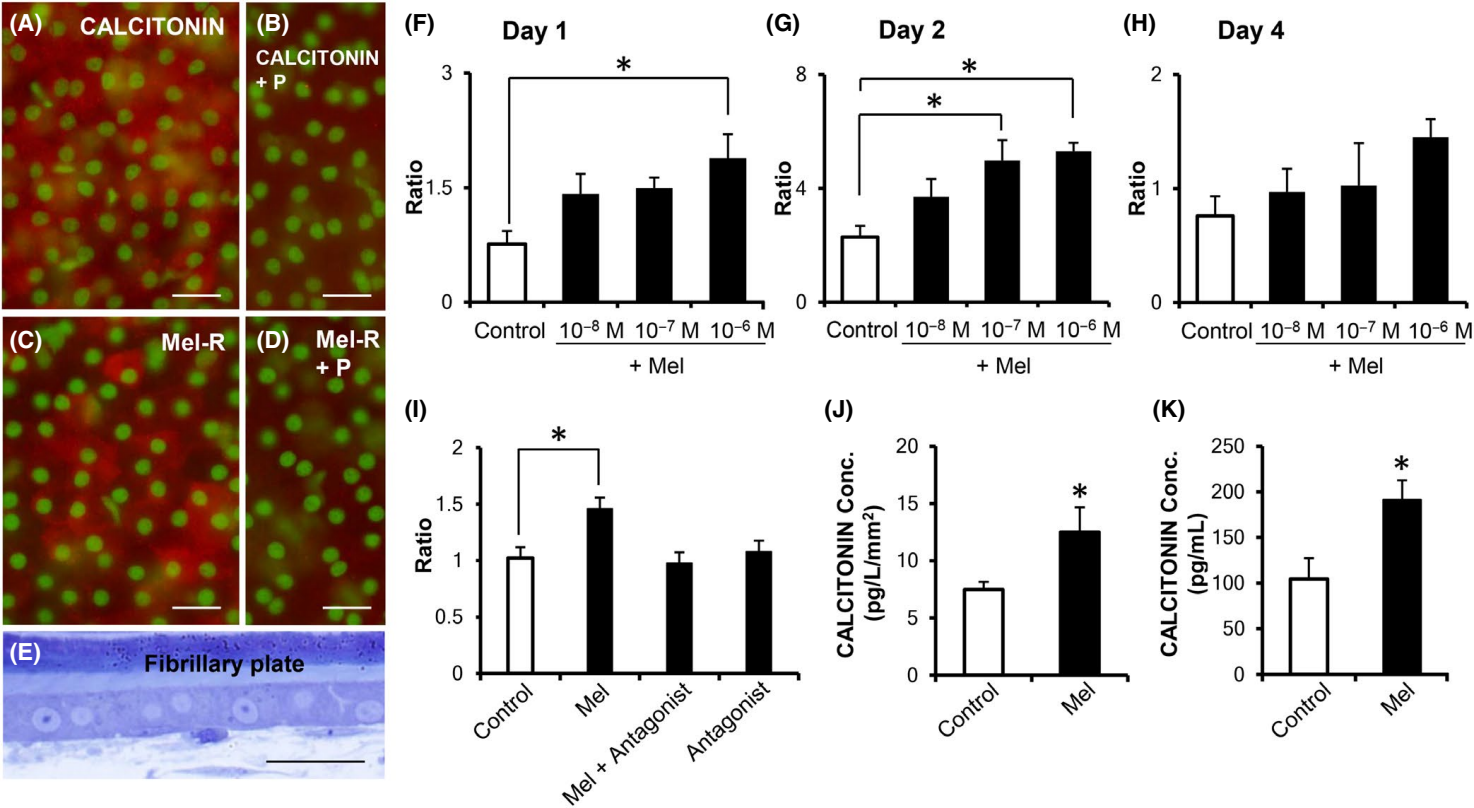
The effect of melatonin on CALCITONIN production in cultured goldfish scales and rat calvariae on the ground. A‐D, Fluorescence microscopy images of the fibrillary plate surface showing endogenous CALCITONIN (red) (A) and melatonin receptor (Mel‐R; red) (C) detected on whole‐mount scales using immunohistochemistry superposed on nuclei stained with DAPI (green). Results of negative controls for CALCITONIN (B) or Mel‐R (D) immune labeling. “+P” means that pre‐absorption was performed with the peptide specific for each antibody. The images are representative data obtained from repeated immunohistochemistry using five or more scales from different goldfish. E, Light microscopy image of the cells on the fibrillary plate observed on a semi‐thin plastic section stained with toluidine blue. Scale bars in A‐E, 20 μm. F‐H, *Calcitonin* expression levels in nontreated (Control) and melatonin‐treated (Mel; 10 nM to 1 μM) regenerating scales cultured for 1 d (F), 2 d (G), and 4 d (H) (n = 7 for each group). I, *Calcitonin* expression levels in scales treated with the Mel‐R antagonist, luzindole. The scales were incubated in the culture medium for nontreated (Control), Mel‐treated (Mel; 1 μM), Mel/luzindole‐treated (Mel + Antagonist; 10 μM, luzindole), or luzindole (10 μM) only‐treated (Antagonist) for 4 d (n = 10 for each group). All targets were standardized to *Ef 1α* expression levels and presented as relative ratios. J, K, CALCITONIN levels measured with ELISA in the culture media of nontreated (Control) and Mel‐treated (1 μM) regenerating scales (n = 10) incubated for 4 d (J) and 2‐d‐old rat calvariae (n = 5) incubated for 1 d (K). The concentrations of CALCITONIN were normalized to the surface areas (mm^2^) of each of the scales. All data are mean ± SEM; *P < .05

CALCITONIN and Mel‐R were immunolocalized in mononuclear cells forming the scale fibrillary plate (Figure 5A‐D). The cells exhibited cuboidal form and cover the surface of the fibrillary plate (Figure 5E). Melatonin treatment significantly increased *Calcitonin* gene expression by days 1 and 2 (Figure 5F‐H). It is well established that melatonin shows its effects by binding to Mel‐R in the plasma membrane to activate intracellular signaling pathways. To test whether melatonin‐induced *Calcitonin* expression is mediated by Mel‐R, we used the Mel‐R antagonist, luzindole, and found that luzindole treatment inhibited melatonin‐induced *Calcitonin* expression (Figure 5I). Validations of housekeeping genes are a crucial component in qPCR analysis, because variation of reference gene expression may cause misinterpretation of the target gene expression level. Accordingly, expression levels of several housekeeping genes, *β‐Actin*, *Gapdh* (*glyceraldehyde 3-phosphate dehydrogenase*), and *Ef1a*, in regenerating scales cultured in dish for 1 day, 2 days, and 4 days were analyzed by qPCR. We then presented raw Ct values for housekeeping genes analyzed (Table S2), as well as expression levels of each housekeeping gene (Figure S7). In the goldfish scales, expression level of *Ef1α* was higher than those of *β‐Actin* and *Gapdh* (Table S2). It was also stable over days in the cultured goldfish scales providing evidence that variation of *Ef1α* expression level does not cause misinterpretation of the target genes' expression in our qPCR analyses. In addition, CALCITONIN levels detected in the culture medium of the scales increased significantly by day 4 (Figure 5J). CALCITONIN production also increased significantly in rat calvariae treated with melatonin for 1 day (Figure 5K).

## 4 DISCUSSION

**Figure 6:**
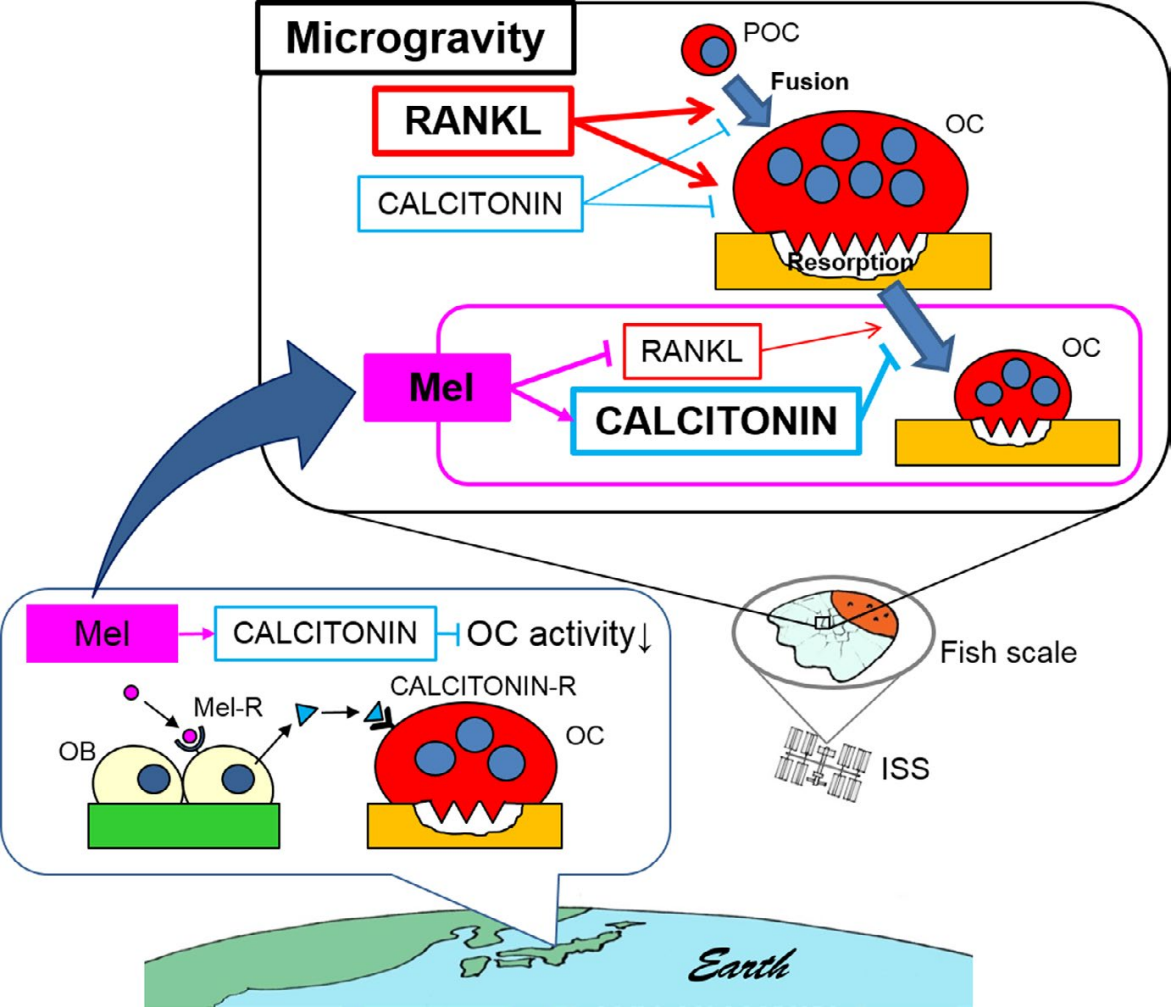
Proposal model. Lower frame: We observed the expression of melatonin receptor (Mel‐R), melatonin synthetic enzymes, and CALCITONIN (an osteoclast‐inhibiting hormone) in osteoblasts in regenerating goldfish scales. Melatonin stimulated CALCITONIN production in the scales. The melatonin‐induced CALCITONIN in turn suppressed osteoclast activity. Upper frame: Microgravity during space flight accelerated the multinucleation and resorption activity in scale osteoclasts, which was associated with the stimulated gene expression of *Rankl* (a major factor for osteoclastogenesis) and the suppressed gene expression of *Calcitonin*. Melatonin administration maintained the normal gene expression levels of the factors during space flight and suppressed microgravity‐stimulated osteoclast activity. The results suggest that melatonin could be used as a prophylactic drug to prevent bone loss in astronauts during space flight. OB, osteoblasts; OC, osteoclasts; POC, preosteoclasts; RANKL, receptor activator of nuclear factor *κ*B ligand; Mel, melatonin; Mel‐R, melatonin receptor; CALCITONIN‐R, CALCITONIN receptor; ISS, the International Space Station

In the experiments based both on the ISS and on the ground, to the best of our knowledge, we are the first to demonstrate a novel melatonin action pathway on bone metabolism using goldfish scales (Figure 6), a bone model with osteoclasts and osteoblasts coexisting on a calcified matrix (Figure S1).

We chose goldfish scales as experimental materials suitable for the analysis of bone metabolism in outer space. Although fish scales and mammalian mineralized skeleton are both derived from dermal skeleton,^28^ there is a long evolutionary gap between them. Fish scales originate from the postcranial dermal skeleton, which has been largely lost in mammals.^29^ In humans, the dermal skeleton is only found as the calvaria and clavicle, which are formed by intramembranous ossification in the same manner as fish scales,^30^ and most of the endoskeleton is formed by endochondral ossification. Nevertheless, fish scales and mammalian mineralized skeleton share many similarities regarding matrix components, cellular morphology, and responses to hormones and mechanical stress.^18^, ^31^, ^32^ In addition, fish scales can be easily obtained in large numbers and are easy to handle as experimental materials.^8^ Therefore, it is considered advantageous to use fish scales as a bone model in experiments and to be able to obtain useful information about the phenomenon occurring in bone tissue under microgravity.

We detected melatonin and melatonin‐synthesizing enzymes in the scale (Figure 1). We also observed that in regenerating scales, melatonin induced *Calcitonin* mRNA expression and CALCITONIN release into media. In addition, Mel‐R was detected in the scale osteoblasts (Figure 5C,D). The results suggest that melatonin increases CALCITONIN production from the osteoblasts via paracrine or endocrine mechanisms to inhibit osteoclast activity in the scales. In our preliminary experiment, melatonin levels in female scales exhibited significant annual variations, with higher levels in April (late reproductive season).^33^ In the reproductive period, the plasma calcium levels in female fish increase remarkably to facilitate production of vitellogenin, which is a major egg protein and a calcium‐binding protein.^34^ In this late period, the osteoclasts in the scales are activated.^35^ We think that melatonin protects scales from excess calcium degradation. Such regulation mechanisms were first reported in goldfish scales. Furthermore, preliminary results showed that melatonin is detected in the culture media of human primary osteoblasts 4 days postincubation Tabuchi Y, Hattori A, and Suzuki N (unpublished data). Human primary osteoblasts also expressed CALCITONIN^36^ and Mel‐R.^37^ These findings, along with our results of culturing rat calvariae (Figure 5K), indicated that melatonin can regulate CALCITONIN production in mammalian bone as well as in goldfish scales.

In the space experiments, we could evaluate resorptive activities using increased groove widths as indicators, and activated osteoclasts in the regenerating scales (Figure 3). Our results are consistent with findings of previous studies analyzing the impact of microgravity on different aspects of osteoclasts.^38^, ^39^ In medaka bone during space flight for 56 days, osteoclast multinucleation also increased, although the total number of nuclei exhibited no significant difference between the ground and flight groups.^38^ The findings support our results, showing that increased resorptive activity in space flight depends on accelerated multinucleation and osteoclast activity but not on increased osteoclast proliferation. A potential explanation for osteoclastic activation in medaka could be the decline in melatonin since *Aanat* and *Asmt* expression levels decreased in the medaka brain including the pineal gland in space (Figure 2B,C).

Microgravity increased the expression ratio of *Rankl*:*Opg* (Figure 4G), a key indicator of bone resorption.^6^ In a space experiment using mice, OPG administration could prevent space flight‐associated increases in bone resorption.^39^ Therefore, the upregulated gene expression ratio of *Rankl*:*Opg* could be due to the activation of osteoclast and bone resorption. Increased expression level of *Cox2a* could facilitate the upregulation of *Rankl*, since COX2 is an isozyme of cyclooxygenase, which is required for the synthesis of prostaglandin E_2_, a stimulator of bone resorption.^40^

Under microgravity conditions, melatonin maintained the gene expression levels detected at F‐1g for *Mmp‐9*, *Ctsk*, *Rankl*, and *Cox2a*, and the *Rankl*:*Opg* ratio at F‐1g (Figure 4A,B,E,G,H). Such conditions also reduced the expression of the osteoclast marker *Rank* (Figure 4D). The results suggest that melatonin suppresses the osteoclast activity under microgravity conditions, potentially via the regulation of the expression of *Rank* in osteoclastic cells and *Rankl* in osteoblastic cells, as above reported in mammalian cells. The suppression of *Rankl* or RANKL by melatonin has been widely reported.^41^, ^42^ Melatonin also tended to increase *Opg* expression in goldfish scales. These effects of melatonin induced the downregulation of the *Rankl*:*Opg* ratio, which should contribute to the suppression of *Rankl*‐mediated osteoclastogenesis. These results were consistent with the previous report that the effects of melatonin were mediated through signaling pathway mediated by Mel‐R, especially its MT2 subtype, on osteoblasts.^42^ Since Mel‐R was detected on scale osteoblasts, these cells should be able to respond to melatonin. In addition, melatonin suppressed *Cox2a* expression (Figure 4H), which is specifically detected in osteoblasts,^40^ indicating the inhibition of synthesis of prostaglandin E_2_, a stimulator of RANKL expression.^43^ Furthermore, microgravity‐dependent induction of osteoblastic markers, *Osx* and *Col1a*, was maintained by melatonin (Figure 4K,L), implying that melatonin could also target specific genes in osteoblasts under microgravity, probably via Mel‐R signaling in scale osteoblasts.

To comprehensively identify the genes whose expression levels were altered in the scales kept in F‐μg condition, we performed next‐generation sequencing analysis (Figure S8). Our analysis found that the F‐μg condition induced the altered expression patterns of osteoclast (*Pth1r* 44 and *Irs1* 45)‐ and osteoblast‐related genes (*Cebpb*,^46^ *Fos*,^47^ *Twist1*,^48^ and *Osr2* 49). These identified genes constituted the networks associated with bone resorption and its formation (Figure S8A,B). Notably, melatonin treatment canceled the alteration of genes' expression (Figure S8C,D). Taken together, these findings provided evidence that melatonin regulates bone resorption and its formation by competing with the microgravity effect on them, consistent with results of the qPCR analysis in Figure 4. Whether microgravity‐induced changes in mRNA expression of analyzed genes translate to an altered expression of the corresponding proteins will be tested in the future.

In the present study, we observed a loss of the melatonin effect on *Calcitonin* expression on day 4 in ground basis experiment (Figure 5H). One possible explanation for this might be a reduction in Mel‐R, as reported by Witt‐Enderby et al^50^ and our previous study.^14^ In fish scales and rat calvariae, on the other hand, we found melatonin‐induced increases in CALCITONIN (Figure 5J,K). This indicates that increase in *Calcitonin* mRNA expression translates to the induced expression of CALCITONIN in fish scales on day 4. Notably, *Calcitonin* expression in the regenerating scales significantly decreased during space flight (Figure 4I). This is the first report of the suppression of *Calcitonin* expression in bone tissues under microgravity conditions. Consistent with our findings, the serum CALCITONIN in monkeys exhibited modest decreases during space flight,^51^, ^52^ while in humans, the blood CALCITONIN levels decreased during 120 days of head‐down tilt bed rest.^53^ Therefore, the suppression of CALCITONIN could play a key role in enhancing bone resorption during space flight. Remarkably, the suppression of *Calcitonin* expression in bone tissues was counteracted by treatment with melatonin (Figure 4I). Although our results strongly indicate that astronauts would benefit from taking melatonin to effectively prevent bone loss, further effort will be required to increase utility of melatonin in the prevention of bone loss. For example, the chemical modification of melatonin would increase its efficacy as a drug preventing bone loss in astronauts.

To further assess the morphological changes taking place in osteoclasts and the mechanism by which melatonin could suppress bone loss during space flight, we cultured the regenerating scales under simulated microgravity conditions created using a 3‐dimensional clinostat (Ground‐µg; Figure S9). After 4 days, the number of nuclei per multinucleated osteoclast (Figure S9G) and the percentage of osteoclasts expressing actin rings (Figure S9H) were higher in scales cultured in Ground‐μg condition than in Ground‐1g condition. The presence of melatonin considerably reduced the impact of simulated microgravity treatment on the ground on both parameters (Figure S9G,H).

Circadian clocks regulate various biochemical, cellular, and physiological processes with a periodicity of approximately 24 hours in order to maximize an organism's physiological efficiency.^54^ Studies have reported molecular links between circadian clocks and bone metabolism in mammals, including humans.^55^ For example, in humans, levels of the C‐terminal cross‐linked telopeptide of type I collagen (a bone resorption marker) and fibroblast growth factor 23 (a regulator of mineral metabolism) display a circadian rhythm.^56^, ^57^ On the basis of these findings, one important issue we raised was whether goldfish scales have circadian clock machinery. In our preliminary experiments, we observed a circadian expression of clock genes in goldfish scales kept under constant light conditions—evidence that goldfish scales have an intrinsic circadian clock. Fish scales have a distinctive utility as experimental systems. They are small and are present on the body surface, facilitating the extraction and analysis of bone components.^9^ Therefore, they are an attractive vertebrate model for examining molecular links between circadian clocks and bone metabolism.

Overall, the present study provides evidence that melatonin suppresses the bone‐resorbing activity of osteoclasts in bone tissues under microgravity conditions via the upregulation of *Calcitonin* and the downregulation of *Rankl* in osteoblasts (Figure 6). In humans, melatonin treatment has been reported to increase bone mineral density^15^ and decreases the bone resorption marker:formation marker ratio in perimenopausal women.^16^ These studies indicated that melatonin can work as an anti‐osteoclastic drug in humans, consistent with our findings. Altogether, we propose melatonin as a potentially promising drug for the prevention of bone loss during space flights.

## AUTHOR CONTRIBUTIONS

MI, MJT, AH, KK, YT, TY, TS, IT, YF, YM, AK, RA, MN, YS, TN, MN, HI, SW, HA, KM, MS, HM, HF, MM, HU, SY, RM, TI, AH, and NS performed experiments. AH, SY, TS, HS, KO, and NS planned the space experiment. ME and TT prepared materials. TT, HN, NS, MN, TK, TH, KH, IT, TY, VSC, KI, TS, SE, YMT, AK, AS, YW, AT, AH, and NS analyzed the data. MI, YF, YT, JH, AH, and NS wrote the manuscript.
