# Supplementary material for: Melatonin is a potential drug for the prevention of bone loss during space flight
Source: J Pineal Res. 2019 Jul 19;67(3):e12594. doi: 10.1111/jpi.12594 (PMC6771646; doi:10.1111/jpi.12594)
Supplement: Supplementary file 3 [file JPI-67-na-s003.pdf]

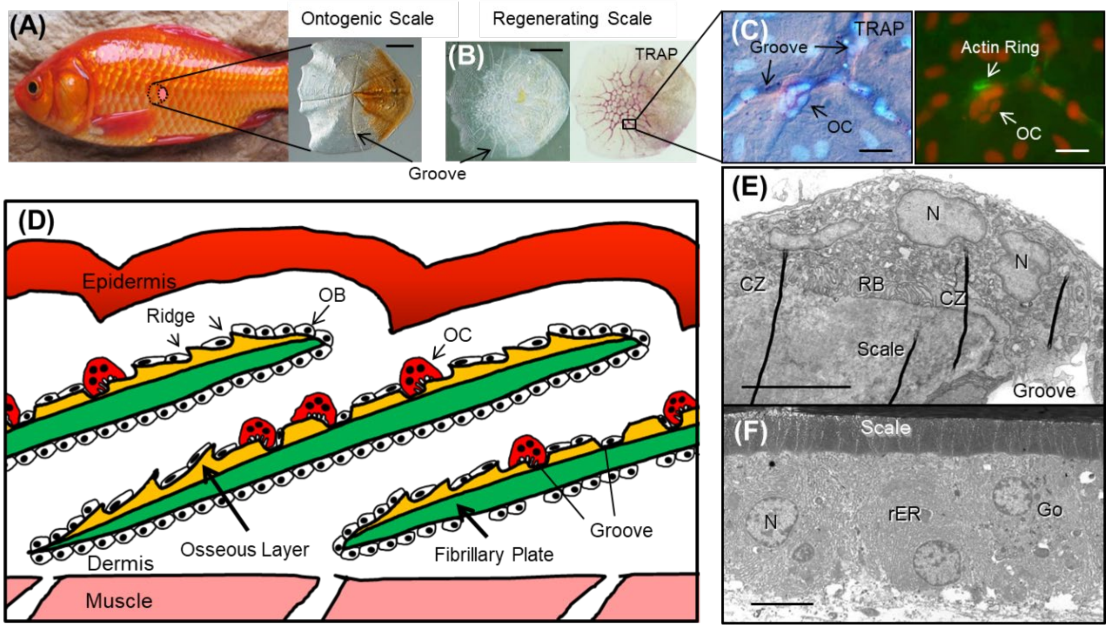

Figure S1 Morphological features of goldfish regenerating scales

(A) A goldfish and a binocular view of an ontogenic scale. Grooves radiate from the center focus. (B) Binocular views of a regenerating scale on day 14 (left) and the same scale stained for TRAP activity, indicated by the red color along the grooves (right). For TRAP staining, goldfish scales were incubated in a solution containing Naphthol AS-BI (0.1 mg/mL; Sigma-Aldrich Co. LLC, St. Louis, MO, USA) as a substrate, Fast Red Violet (0.7 mg/mL; Sigma-Aldrich Co. LLC) as a diazonium coupling salt, and 50 mM tartrate. The grooves formed a mesh-like structure at the center of the goldfish scales. (C) Light microscopic views of the surface on the osseous layer of the regenerating goldfish scales cultured for 86 h at 22°C on the ground. TRAP staining (left) and F-actin staining (right) superposed with DAPI staining of the nuclei. For F-actin staining, goldfish scales were stained using 1% Alexa Fluor® 488 phalloidin (Molecular Probes, Eugene, OR, USA) in phosphate buffer solution for 4 days at 4°C in the dark. A TRAP-positive multinucleated osteoclast possessed an actin ring along the groove. (D) Schematic illustration of regenerating scales. (E) Electron microscopic image of a representative osteoclast on the osseous layer of goldfish scales on the ground. On the groove edge of the goldfish scales, a multinucleated osteoclast exhibited a well-developed RB and CZs. (F) Electron microscopic image of osteoblasts on the fibrillary plate. They exhibit a well-developed rER and Go. OB, osteoblast; OC, osteoclast; N, nucleus. Scale bars = 1 mm in A and B, 10 μm in C, and 5 μm in E and F. TRAP, tartrate-resistant acid phosphatase; DAPI, 4', 6-diamidino-2-phenylindole; RB, ruffled border; CZ, clear zone; rER, rough endoplasmic reticulum; Go, Golgi apparatus.

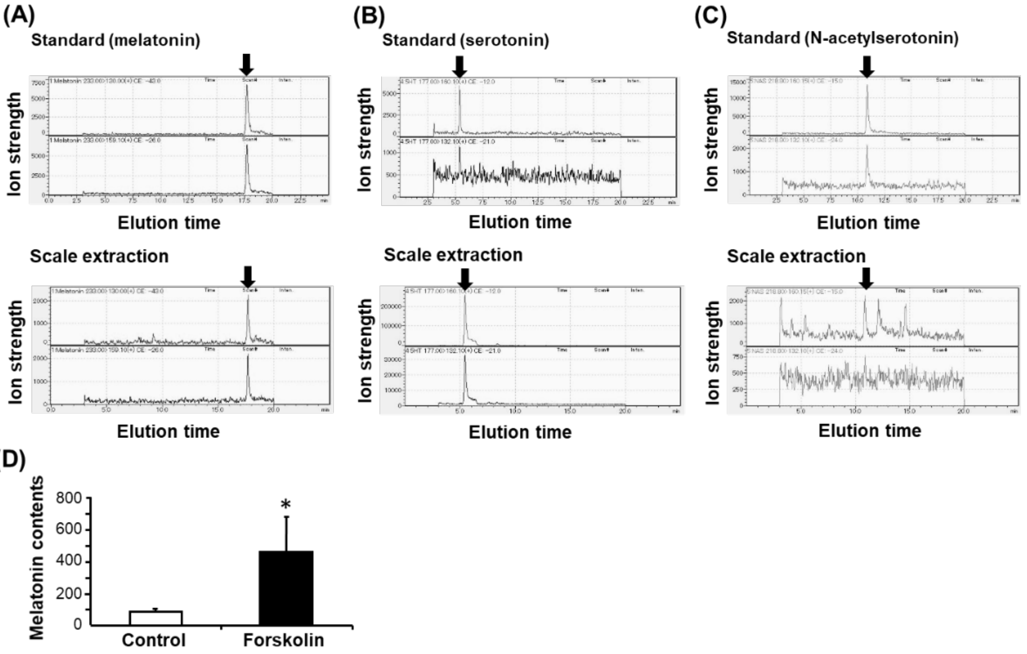

Figure S2 Detection of melatonin and its precursors in goldfish scales

Melatonin and its precursors in goldfish scales were detected using the LCMS-8050 Triple Quadrupole LC-MS/MS system (Shimadzu, Kyoto, Japan): (A) melatonin, (B) serotonin, and (C) N-acetylserotonin. Goldfish were kept under 300 lux light conditions in a 12-h:12-h light–dark cycle (light turned on at 8:00 a.m.) at 26°C. Scales were collected from the goldfish under anesthesia with 0.03% ethyl 3-aminobenzoate and methane sulfonic acid salt (MS-222; Nacalai Tesque, Kyoto, Japan) neutralized with 0.03% sodium bicarbonate. After sonication and acetone extraction, the goldfish scale samples were centrifuged at 1000 × *g*, and supernatants were evaporated to dryness using N_2 gas. The extracts were redissolved in 100 µL of distilled water and filtered through a PVDF 0.22 µm centrifugal filter unit (Merck Millipore, Darmstadt, Germany). Next, 10 µL of this solution was used for LC-MS/MS analysis. A 2.0 mm × 150 mm, 3.0-µm particle size ODS column (Tosoh Bioscience LLC, King of Prussia, PA, USA) was used at a column oven temperature of 25°C. The flow rate of the mobile phase was 0.3 mL/min. A gradient was used, with mobile phase A being 10 mM ammonium acetate with 0.05% acetic acid and mobile phase B being 100% methanol. The gradient started with 5% mobile phase B and increased to 50% mobile phase B within 20 min. (A–C) The arrows indicate the respective peaks of melatonin (A), serotonin (B), and N-acetylserotonin (C) in the upper (standard) and lower (scale extraction) panels. (D) Goldfish scales were incubated with or without forskolin (10 µM) for 18 h, after which the incubation the melatonin contents were measured using LC-MS/MS. Data are presented as the mean ± SEM; $n = 8$; *$P < 0.05$. LC-MS/MS, liquid chromatography tandem mass spectrometry; PVDF, polyvinylidene difluoride; ODS, octadecylsilyl; SEM, standard error of the mean.

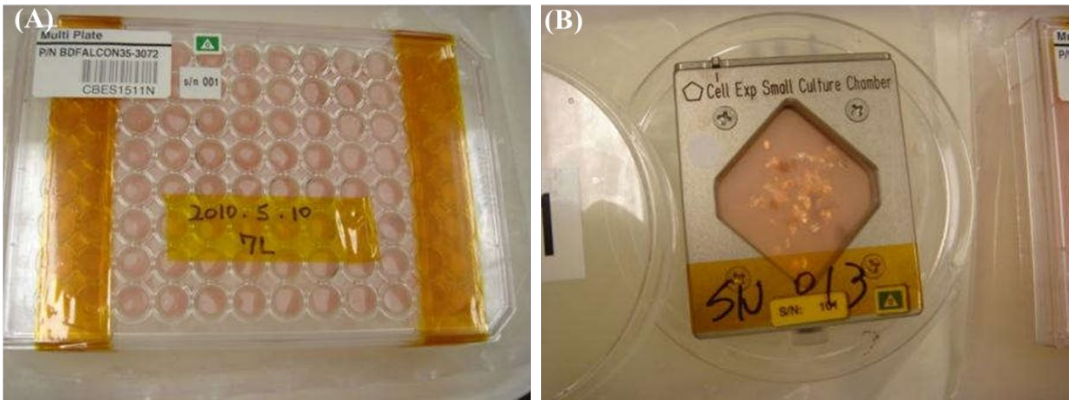

Figure S3 Preparation of regenerating goldfish scales for the experiments in outer space

The goldfish were anesthetized, and their scales were removed one by one in two horizontal, bilateral lines using sharpened forceps. Subsequently, the goldfish were kept under 300 lux light conditions with a 12-h:12-h light–dark cycle (light turned on at 8:00 a.m.) at 26°C. On day 12 after removal of their scales, the goldfish were bred in water with the anti-infection reagent F Gold (Japan Pet Design Co. Ltd., Tokyo, Japan). The next day, a few regenerating scales were collected from the goldfish to measure alkaline phosphatase for osteoblast activity and TARP for osteoclast activity, and 14 individual goldfish with similar cell activities were selected from the 40 goldfish prepared, as described before. On day 14, regenerating scales were sampled from goldfish on ice that had been sterilized using hypochlorous and fungizone solutions and immersed in Leibovitz's L-15 medium (Invitrogen, Grand Island, NY, USA) containing 10% FCS (Nichirei Biosciences, Inc., Tokyo, Japan), 100 U/mL of penicillin, 100 μg/mL of streptomycin, and 200 μg/mL of kanamycin. Sterilized goldfish scales were packed into 96-well plates (1 scale/per well) (A) and Cell Exp Small Chambers (Chiyoda Corporation, Yokohama, Japan) (60 scales/chamber) (B) with or without melatonin (1 μM) and stored for 4 days at 4°C before space shuttle STS-132 (ULF4) was launched to the ISS. TRAP, tartrate-resistant acid phosphatase; FCS, fetal calf serum; ISS, International Space Station.

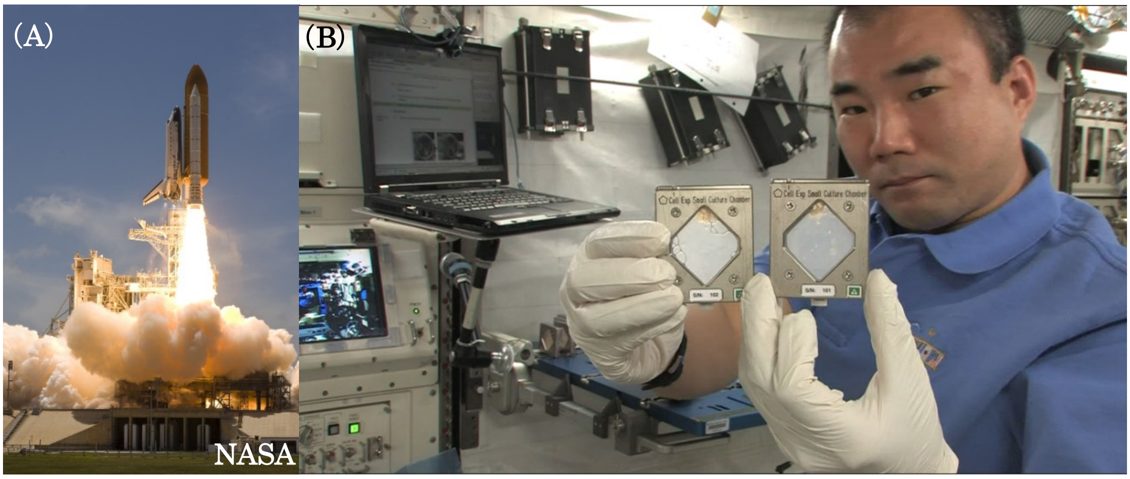

Figure S4 Photographs of the space shuttle launch scene and onboard experiment

Space shuttle STS-132 (ULF4), which carried the specimens, was launched to the ISS on May 14, 2010 (A). JAXA performed the experiments using the Measurement Experiment Unit installed in the CBEF on-board Kibo (B). JAXA astronaut Soichi Noguchi at work in the ISS, holding Cell Exp Small Chambers (Chiyoda Corporation) containing goldfish scales (see supplemental movies). After arrival at the ISS, 96-well plates and culture chambers were incubated for 86 h at 22°C under microgravity using the CBEF and compared with a 1-g control in space. After the experiments, goldfish scales in 96-well plates were frozen at −95°C for cell activity analysis. The goldfish scales packed into culture chambers were used to conduct morphological or gene expression analyses. Goldfish scales for morphological analysis were preserved in 4% paraformaldehyde in phosphate buffer solution. For mRNA expression analysis, the goldfish scales were preserved with RNA-later (Nippon Gene Co., LTD., Tokyo, Japan) and then frozen. Cold storage samples were installed in a Laboratory Freezer for ISS in the refrigerator or freezer compartments. On May 26, 2010, the specimens cultured in Kibo were returned to Earth by space shuttle STS-132 and analyzed for biological characteristics. JAXA, Japan Aerospace Exploration Agency; CBEF, Cell Biology Experiment Facility; ISS, International Space Station; mRNA, messenger RNA.

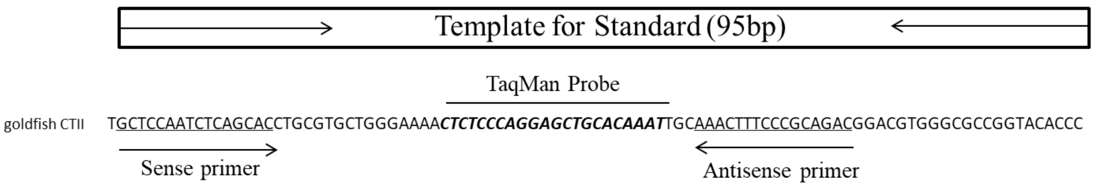

Figure S5 Location of primers (TaqMan probe and Standard) and TaqMan probe for goldfish *Calcitonin*

To analyze the expression of *Calcitonin* mRNA in regenerating goldfish scales, the TaqMan probe was designed. The location of primer sets and the TaqMan probe in the genomic sequence of goldfish *Calcitonin* are shown. The primers and the probe were synthesized as described previously (Suzuki, Ueda, Sakamoto, & Sasayama, 1999). A relative quantification was performed using the standard curve method according to the manufacturer's instructions. The stocked cDNAs were accurately diluted. Standard curves were prepared using the diluted cDNAs. The relative quantities determined could be compared across plates. The condition for PCR amplification was 45 cycles of denaturation for 10 s at 95°C and annealing for 40 s at 58°C. The mRNA expression levels were normalized to the *EF1α* mRNA level. *EF1α* was selected as a gene whose expression level was higher than expression levels of *β-Actin* and *Gapdh* (Table S2) and fluctuation level was low (Figure S7). *EF1α* expression level did not change among the ground (0.1402 ± 0.0079), F-1g (0.1456 ± 0.0053), and F-μg (0.1463 ± 0.0022) conditions and did not decrease after culture, because the culture was performed with a medium containing FCS. mRNA, messenger RNA; cDNA, complementary DNA; PCR, polymerase chain reaction; EF1α, elongation factor-1 alpha; Gapdh, glyceraldehyde 3-phosphate dehydrogenase; F-1g, flight artificial 1g; F-μg, flight microgravity; FCS, fetal calf serum.

Reference

Suzuki N, Ueda K, Sakamoto H, & Sasayama Y. (1999). Fish calcitonin genes: Primitive bony fish genes have been conserved in some lower vertebrates. *Gen Comp Endocrinol*, 113, 121-127. doi: 10.1006/gcen.1998.7215

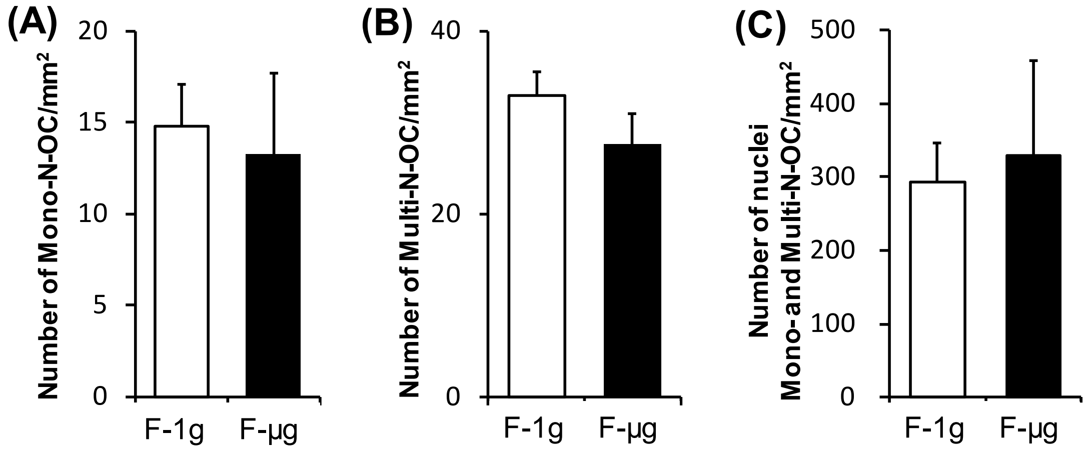

Figure S6 Cell number and total nuclear number of osteoclasts

The regenerating goldfish scales cultured in space were selected randomly and histomorphometric analyses were performed in 6 views of 0.31-mm^2 observation areas under a microscope with a 10× objective lens. Mean measurements from 6 views were regarded as representative values for each scale. TRAP-positive cells were identified as osteoclasts. (A) Cell number of mononucleated osteoclasts per mm^2 on goldfish scales of F-1g or F-μg; P = 0.37. (B) Cell number of multinucleated osteoclasts per mm^2 on goldfish scales of F-1g or F-μg; P = 0.11. (C) Total number of nuclei in mononucleated and multinucleated osteoclasts per mm^2 on goldfish scales of F-1g or F-μg; P = 0.24. Data are presented as the mean ± SEM (n = 6 for F-μg and 8 for F-1g). TRAP, tartrate-resistant acid phosphatase; F-μg, flight microgravity; F-1g, flight artificial 1g; SEM, standard error of the mean.

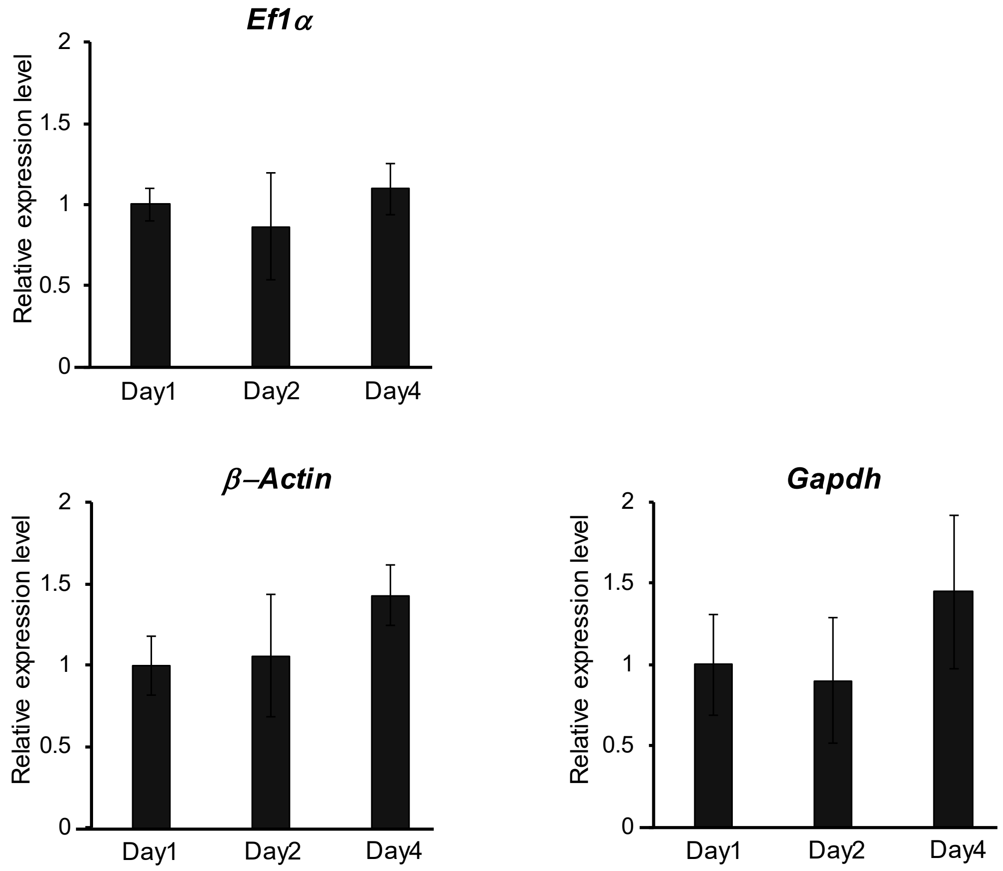

Figure S7 Expression profiles of housekeeping genes in regenerating goldfish scales

Expression levels of *β-Actin*, *Gapdh*, and *Ef1α* in regenerating scales cultured in dish for 1 day (Day 1), 2 days (Day 2), and 4 days (Day 4) were analyzed by qPCR. The value at Day 1 was set as 1 for each gene. Values are mean ± SEM of seven independent experiments. Gene-specific primers for *β-actin* (sense: 5'-CGAGCGTGGCTACAGCTTCA-3'; antisense: 5'-GCCCGT-CAGGGAGCTCATAG-3') (AB039726), *Gapdh* (sense: 5'-CGCCGATGTGTCTGTTGTTG-3'; antisense: 5'-CTTGACGGCCTCCTTGATGT-3') (FX986058), and *Ef1α* (sense: 5'-ATTGTTGCTGGTGGTGTTGG-3'; antisense: 5'-GGCACTGACTTCCTTGGTGA-3') (AB979720) were used. The condition for PCR amplification was 40 cycles of denaturation for 10 s at 95°C and annealing for 40 s at 60°C. EF1α, elongation factor-1 alpha; Gapdh, glyceraldehyde 3-phosphate dehydrogenase; SEM, standard error of the mean.

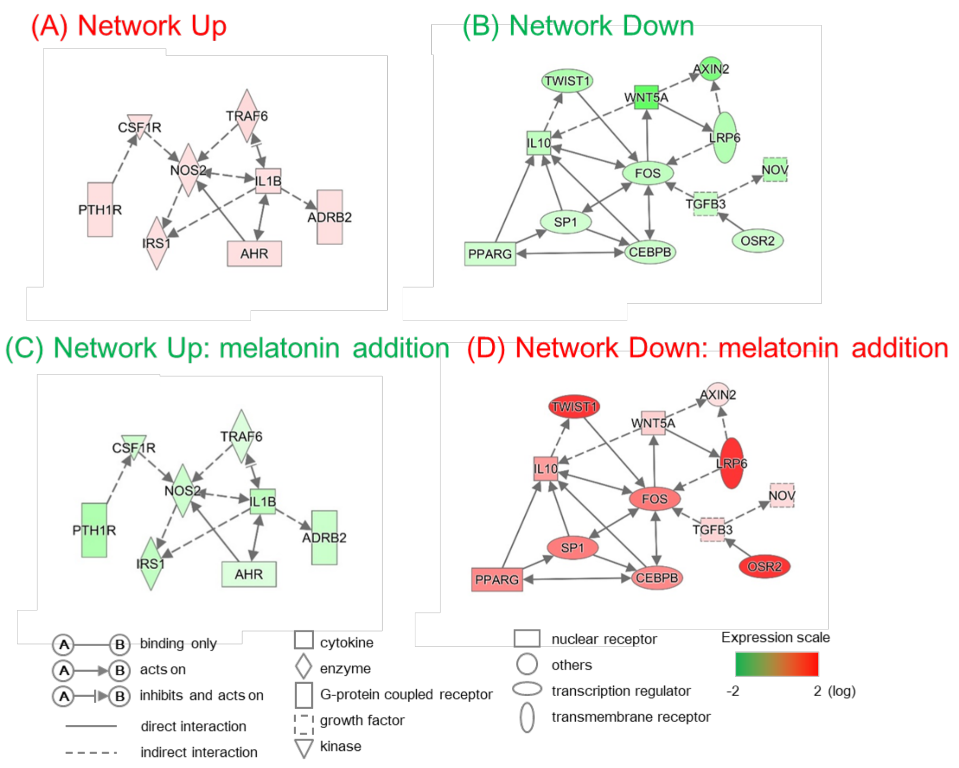

Figure S8 Effects of melatonin on the gene expression under F-μg in regenerating goldfish scales

RNA-seq libraries for directional paired-end reads (100-bp paired end) were constructed from the scale mRNAs using the TruSeq RNA Sample Prep Kit v2, and subjected to sequencing analysis with HiSeq 2000 (Illumina, San Diego, CA, USA) and cluster generation. Quality control was performed for the raw reads using FastQC (http://www.bioinformatics.babraham.ac.uk/projects/fastqc/), and high-quality reads (> Q20 and > 36 bp) without adaptors were extracted using Trimmomatic (Bolger, Lohse, & Usadel, 2014). Next, a *de novo* transcriptome assembly was conducted with the filtered reads using Trinity with default parameters to establish the reference sequence of goldfish transcripts (Haas et al., 2013), and sequence reads were mapped to the reference sequence using Bowtie2 with the very sensitive local parameter; the mapping rates were >98% (Langmead & Salzberg, 2012). The assembled sequences were also annotated using Blastx and Blastn against the NCBI database, and the mapped read counts with annotation were normalized using the Fragments Per Kilobase of exon per Million mapped reads method. The data were further analyzed using GeneSpring software (Agilent Technologies, Santa Clara, CA, USA) to extract significant genes. To examine gene ontology, including biological processes, cellular components, molecular functions, and gene networks, the obtained data were analyzed using Ingenuity® Pathway Analysis tools (Qiagen, Venlo, the Netherlands) (Tabuchi et al., 2006). (A and B) Networks of genes that were expressed

differentially in goldfish scales cultured in F-μg were extracted by analysis using Ingenuity Pathway analysis software. Network up (A) and network down (B). (C and D) Effect of melatonin on the expression levels of genes. Some goldfish scales were cultured using 1 μM melatonin during the space flight. Expression levels of genes in Network up (C) and Network down (D) were down- and up-regulated in goldfish scales cultured in F-μg with melatonin, respectively.

The network was displayed graphically as nodes (genes) and edges (biological relationships between nodes). Nodes and edges displayed various shapes and labels that present the functional class of genes and the nature of the relationship between nodes, respectively. RNA-seq, RNA-sequencing; F-μg, flight microgravity; F-1g, flight artificial 1g; NCBI, National Center for Biotechnology Information.

References

Bolger AM, Lohse M, & Usadel B. (2014) Trimmomatic: A flexible trimmer for Illumina sequence data. *Bioinformatics* 30, 2114-2120. doi: 10.1093/bioinformatics/btu170

Haas BJ, et al. (2013) De novo transcript sequence reconstruction from RNA-seq using the Trinity platform for reference generation and analysis. *Nat Protoc* 8,1494-1512. doi: 10.1038/nprot.2013.084.

Langmead B & Salzberg S. (2012) Fast gapped-read alignment with Bowtie 2. *Nat Meth* 9, 357-359. doi: 10.1038/nmeth.1923.

Tabuchi Y et al. (2006) Genetic networks responsive to sodium butyrate in colonic epithelial cells. *FEBS Lett* 580, 3035-3041. doi: 10.1016/j.febslet.2006.04.048.

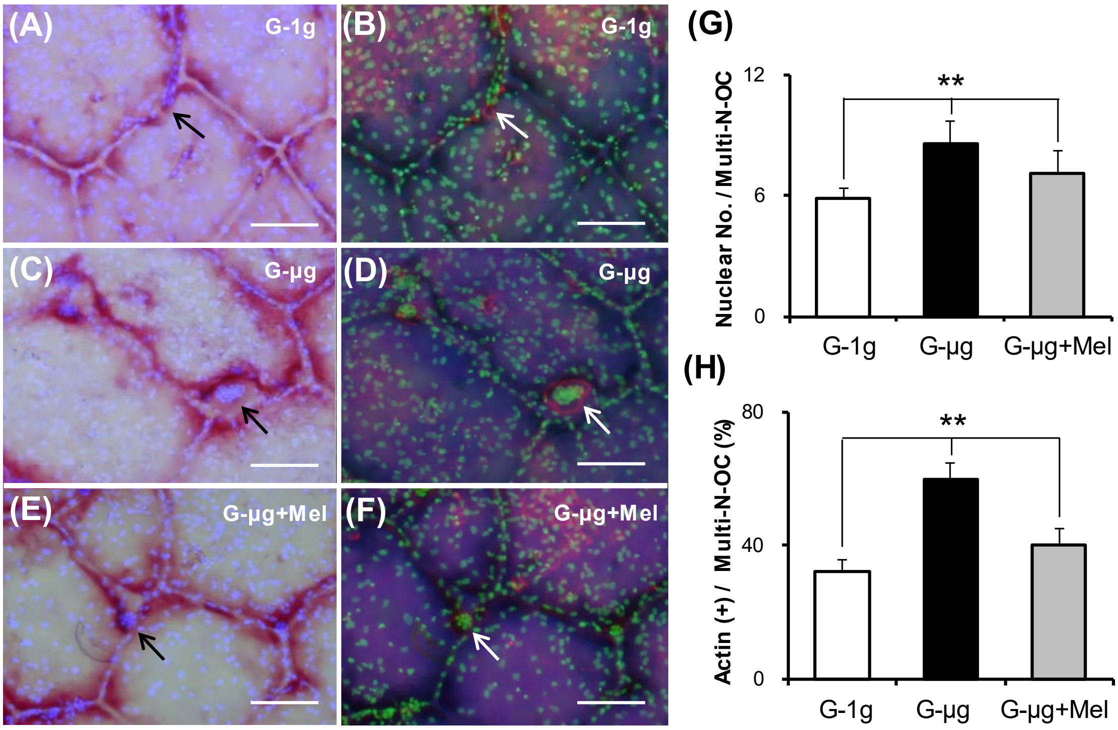

Figure S9 Morphological analysis of osteoclasts in regenerating goldfish scales cultured in simulated microgravity on the ground

Goldfish scales having regenerated for 14 days were cultured in 96-well plates in simulated microgravity with a 3D clinostat (Chiyoda Corporation) on the ground for 4 days at 22°C. The conditions of 3D clinostat were described previously (Hirasaka et al., 2005). As a control, regenerating goldfish scales were cultured in static conditions on the ground at the same time. The goldfish scales were fixed and prepared for morphological analysis as in the space experiment (A–F) Regenerating goldfish scales cultured for 4 days in static conditions (ground 1g: G-1g) (A and B) and clinostat-simulated microgravity (ground µg: G-µg) without (C and D) and with melatonin (1 µM) (E and F). The goldfish scales were fixed and stained for TRAP (A, C, and E) or actin rings (B, D, and F). Multinucleated cells exhibited intense TRAP activity (black arrows) and actin rings (white arrows). Scale bar = 100 µm. (G) Average number of nuclei per multinucleated osteoclast (n = 5). (H) Percentage of multinucleated osteoclasts with actin rings (n = 5). Data are presented as the mean ± SEM; $^{**}P < 0.01$. TRAP, tartrate-resistant acid phosphatase; SEM, standard error of the mean.

Reference

Hirasaka K et al. (2005) Clinorotation prevents differentiation of rat myoblastic L6 cells in association with reduced NF-κB signaling. *Biochim. Biophys. Acta*, 1743, 130–140. doi:10.1016/j.bbamcr.2004.09.013

Table S1 Sequences of primers used for quantitative real-time PCR analysis

Name	Forward primer	Reverse primer	Accession No.
Aanat	CCGCTAAGTGTTGCCAGTCCTC	CGTGTTTTCTCATCGCTTCCTGTTCT	AB274853
Asmt	GGAGGCTGCTCTGGTGCATT	GGTGTCGTCTTGCTGGGTGA	AB274851
*Mmp*9	GCTTCTGCCCCAGTGAGCTT	GTGGAGCACCAGCGATACCC	AB889498
Ctsk	TGGGAGGGCTGGAAACTCAC	CATGAGCCGCATGAACCTTG	AB236969
Trap	AACTTCCGCATTCCTCGAACAG	GGCCAGCCACCAGGAGATAA	AB236968
Rank	GGGAGATGCTGCGAAAAATG	TTTAGGGTTGTGTGGACGAGTG	AB894121
Rankl	CGAGTGTGGCGATTTTGTTG	ATGGGCGTCTTGATTGGAAG	AB894120
Opg	CGTGAACACGGTGTGCGAGTGT	CCTCTGCGCAGGCCTCACA	AB970727
Cox2a	ACTTGTCCTATTACACCCGCACCC	GGTGAGAGAAATGCTGGGCGAAGA	LC107514
Runx2b	GACAGCAGCAGCAGGACAGC	TGAGAAGGCAACACCGAGCA	AB274887
Osx	GACTGCCTGACCAGCGTCAA	GAGGCACCAAGCCTCTCCAA	AB274888
Col1a	TGCAACCAGGATGCCATCAA	ATGAGGCGCAGGAAGGTGAG	AB685219
Ocn	ATGCCTGAGCGCAGGTCTTC	CACAGGCCAGGTTTGCTTCA	AB685220
Ef1α	ATTGTTGCTGGTGGTGTTGG	GGCACTGACTTCCTTGGTGA	AB979720

Primer sets for *Aanat*, *Asmt*, *Mmp9*, *Ctsk*, *Trap*, *Rank*, *Rankl*, *Opg*, *Cox2a*, *Runx2b*, *Osx*, *Col1a*, *Ocn*, and *Ef1α* are presented. PCR, polymerase chain reaction; *Aanat*, *aralkylamine N-acetyltransferase*; *Asmt*, *acetylserotonin O-methyltransferase*; *Mmp9*, *matrix metalloproteinase-9*; *Ctsk*, *cathepsin K*; *Trap*, *tartrate-resistant acid phosphatase*; *Rank*, *receptor activator for nuclear factor κB*; *Rankl*, *receptor activator for nuclear factor κB ligand*; *Opg*, *osteoprotegerin*; *Cox2a*; *cyclooxygenase-2a*; *Runx2b*, *runt-related transcription factor 2b*; *Osx*, *osterix*; *Col1a*, *type 1 collagen 1a*; *Ocn*, *osteocalcin*; *Ef1α*, *elongation factor 1α.*

Table S2 Raw Ct values for housekeeping genes analyzed by quantitative real-time PCR

Day	Sample #	*Ef1α*	*β-Actin*	*Gapdh*
1	1	15.81	20	21.29
	2	15.72	19.88	21.91
	3	15.74	19.85	21.06
	4	15.71	19.82	20.89
	5	16.13	20.62	21.87
	6	15.85	20.18	21.27
	7	16.08	20.33	22.05
	Ave ± SEM	15.86 ± 0.17	20.09 ± 0.29	21.47 ± 0.45
2	1	16.47	20.43	21.65
	2	15.86	20.1	22
	3	16.35	19.96	21.62
	4	16.06	19.8	22.31
	5	15.3	19.25	20.8
	6	16.9	21.08	22.32
	7	16.09	19.92	21.03
	Ave ± SEM	16.14 ± 0.50	20.07 ± 0.56	21.67 ± 0.59
4	1	15.65	19.44	20.09
	2	15.83	19.58	20.89
	3	15.37	19.24	20.2
	4	15.78	19.86	20.8
	5	15.85	19.67	21.71
	6	15.94	19.59	20.84
	7	15.65	19.44	21.33
	Ave ± SEM	15.72 ± 0.18	19.54 ± 0.19	20.83 ± 0.57

Expression levels of *Ef1α*, *β-Actin*, and *Gapdh* in regenerating scales cultured in dish for 1 day (Day 1), 2 days (Day 2), and 4 days (Day 4) were analyzed by qPCR as described in Figure S7. EF1α, elongation factor-1 alpha; Gapdh, glyceraldehyde 3-phosphate dehydrogenase; Ave, average; SEM, standard error of the mean; qPCR, quantitative real-time PCR.

Movie Captions

Movie S1 Astronauts handling biospecimens under microgravity in the International Space Station

Japan Aerospace Exploration Agency (JAXA) astronaut Soichi Noguchi removed the culture medium from culture chambers with a Pre-Fixation Kit. Thereafter, he removed the culture chambers from the Pre-Fixation Kit.

Movie S2 Preparation of fixative treatment for biospecimens in culture chambers

JAXA astronaut Soichi Noguchi removed the lid of the Cell Fixation Kit before inserting the culture chamber into it. The medium in the culture chamber was removed, as shown in Movie S1. Using the Cell Fixation Kit, the cultured goldfish scales in the culture chamber were fixed with neutralized 4% paraformaldehyde. The culture chamber was floating while he carefully checked the procedure displayed on the laptop.

Methods for histomorphometry

Observed areas on the regenerating goldfish scales

For the space flight experiment, 6 (for F-μg) or 8 (for F-1g) goldfish scales were selected randomly for histomorphometry analyses (Figure 3). For the clinostat experiment, 5 goldfish scales were selected randomly for each group from the 10 goldfish scales used for the experiment (Figure S9). Analysis was performed in 6 views of 0.31-mm^2 observation areas under a microscope with a 10× objective lens. Mean measurements from 6 views were regarded as representative values for each goldfish scale.

Groove width

The width at the middle of each groove in the observed area was measured using National Institutes of Health (NIH) ImageJ software, and the average width of each goldfish scale was determined.

Number of osteoclast nuclei

All specimens examined by histomorphometry were stained for tartrate-resistant acid phosphatase (TRAP) activity. TRAP-positive cells in the observed area were counted as osteoclasts, while TRAP-positive cells with three or more nuclei were identified as multinucleated osteoclasts. The mean number of nuclei per multinucleated osteoclast was also calculated. Osteoclast boundaries were confirmed using hematoxylin staining.

Actin ring–positive osteoclasts

The percentage of osteoclasts with actin rings in the observed area was determined for each goldfish scale.

Actin ring sizes and actin ring–covered groove edges

The sizes of the actin rings were determined by their lengths along the grooves in the observed area. The percentage of groove lengths covered with actin rings and the total groove lengths were measured using NIH ImageJ software, and the percentages of actin ring–covered groove lengths were determined.
